# Encoding Praise and Criticism During Social Evaluation Alters Interactive Responses in the Mentalizing and Affective Learning Networks

**DOI:** 10.3389/fnins.2018.00611

**Published:** 2018-09-04

**Authors:** Shan Gao, Yayuan Geng, Jia Li, Yunxiao Zhou, Shuxia Yao

**Affiliations:** ^1^School of Foreign Languages, University of Electronic Science and Technology of China, Chengdu, China; ^2^The Clinical Hospital of Chengdu Brain Science Institute, MOE Key Laboratory for NeuroInformation, University of Electronic Science and Technology of China, Chengdu, China

**Keywords:** praise, criticism, social inference, the mentalizing network, affective learning

## Abstract

Verbal communication with evaluative characters of different emotional valence has a considerable impact on the extent to which social relations are facilitated or undermined. Here using functional magnetic resonance imaging, we investigated how the brain acts in response to social praise and criticism, leading to differential affective judgments. We engaged thirty men and women in a task associating sex-balanced, neutral faces with praising or criticizing comments targeting others or objects. A whole-brain analysis revealed that criticism as compared to praise enhanced the activation in the medial prefrontal cortex (mPFC), particularly its dorsal portion, whereas the right amygdala displayed an opposite pattern of changes. Comments on others relative to objects increased the reactivity in the left posterior superior temporal sulcus and posterior cingulate cortex (PCC) such that both praise and criticism of others produced stronger activation in these two regions than their object-targeted counterparts. The interaction of valence and target was identified in the mPFC with greater reactivity in the contrasts of criticism vs. praise in the social context and others- vs. object-targeted criticism. Comments also modulated the functional connectivity of prior activated regions with the left temporoparietal junction, bilateral caudate and left PCC/precuneus showing reduced connectivity in response to social criticism but greatly strengthened connectivity for social praise as compared to non-social counterparts. These neural effects subsequently led to altered likeability ratings for the faces. Neither behavioral nor neural effects observed were influenced by the gender of participants. Taken together, our findings suggest a fundamental interactive role of the mentalizing and affective learning networks in differential encoding of individuals associated with praising or criticizing others, leading to learning of valenced traits and subsequent approach or avoidance responses in social interactions.

## Introduction

Language has been proposed to function as a context that shapes human perception (Barrett et al., 2007). When an individual talks, verbal information conveying different affective value may promote inference of character traits, affect how this person is judged (Bliss-Moreau et al., 2008; Baron et al., 2011; Schwarz et al., 2013), and thus alter the balance between approach and avoidance behavior (Todorov et al., 2008). In everyday communication, those who tend to criticize often may display their negative (e.g., anti-social) attributes and lead to others disfavoring and avoiding them socially; in contrast, praising comments may play the opposite role (Blair et al., 2008; Gao et al., 2016; Miedl et al., 2016). To facilitate favorable social impressions and interactions, it is of great importance to examine how people respond neurally and behaviorally to a person’s criticizing and praising. In particular, it is important to distinguish the impact of verbal comments made concerning others rather than objects, since previous research suggests a stronger impact of social relative to non-social contexts (Bliss-Moreau et al., 2008; Anderson et al., 2011; Gao et al., 2016).

Learning or making inferences about other people including their intentions, beliefs, and traits implicates a brain network supporting mentalizing (Mitchell, 2009; Van Overwalle, 2009; Ma et al., 2011). A wealth of evidence has shown that this network comprises the medial prefrontal cortex (mPFC), including its dorsal portion, posterior cingulate cortex (PCC) or precuneus, temporoparietal junction (TPJ) and posterior superior temporal sulcus (pSTS) (Frith and Frith, 2006; Mitchell, 2009; Muscatell et al., 2012). Here we associated neutral faces of different individuals with praising and criticizing comments to cue social inferences and thus hypothesized that the mentalizing network would be activated and modulated by encoding of verbal praise and criticism. We also hypothesized that the mentalizing network might respond differentially to person- and object-directed comments since this neural circuitry appears sensitive to how person-related the judgment context is (Mitchell et al., 2002). In the mentalizing network, the mPFC was our primary candidate region given that it has been suggested to be tuned to social valence (Harris et al., 2007). However, we did not have further predictions on whether the responses of this region would be more intense to social criticism or to social praise since previous findings in this regard remained contradictory such that some showed stronger responses to negative (Perry et al., 2012) whilst some to positive social information (Harris et al., 2007).

Verbal comments in our paradigm contain emotional properties, which may give rise to the recruitment of the amygdala. This region is sometimes involved in mentalizing tasks due to its essential role in emotion processing (Mitchell et al., 2005). It has also been implicated in processing positive and negative emotional value assigned to neutral agents via associative learning (Everitt and Robbins, 1992; Phelps and LeDoux, 2005; Schiller et al., 2009). Given the differentiated emotional and motivational values of praise and criticism in our social judgment context we were therefore interested in assessing how amygdala responses would be modulated and how these responses would interact with those in the mentalizing network.

Gender differences in social behavior have long been postulated (Eagly, 1987) but not yet well established in the use of verbal cues for social inferences. However, they have been reported in understanding others by young children (Dunn et al., 1991) and in the activity of neural correlates of mentalizing (Krach et al., 2009). We therefore exploratorily investigated whether men and women would encode praise and criticism differently, leading to divergent affective judgments.

## Materials and Methods

### Participants

Thirty healthy Chinese participants (15 males; age range, 20–25; *M* ± SD, 22.73 ± 1.57 years) were recruited by local advertisement. All participants were right-handed, had no vision problems or language disabilities, and reported no history of neurological or psychiatric disorders. Participants with MRI-contraindications were excluded from participation. The experiment had full ethical approval from the local ethics committee at the University of Electronic Science and Technology of China and all participants gave written informed consent in accordance with the latest revision of the Declaration of Helsinki.

### Stimuli

Our paradigm used facial pictures of 36 individuals (balanced for sex) and 144 verbal comments of the same length (in Chinese). In a pre-test incorporating an independent sample (*n* = 36, 18 males) all faces were rated as emotionally neutral (*M* ± SD, 4.985 ± 0.149) and average in attractiveness (4.378 ± 0.351) and trustworthiness (4.871 ± 0.324) using 9-point Likert scales. All comments in the four categories (criticizing-others/praising-others/criticizing-objects/praising-objects) were also assessed by an independent sample (*n* = 30, 15 males). A two-way repeated-measures analysis of variance (ANOVA) showed that criticizing and praising comments significantly differed in terms of valence (*F*_1,29_ = 144.11, *P* < 0.001, $\eta_{p}^{2}$ = 0.832); no other valence differences were found. Moreover, the four categories of comments did not differ in arousal, likelihood and comprehension ratings (all *P*s > 0.1; **Supplementary Table S1**).

### Procedure

**FIGURE 1:**
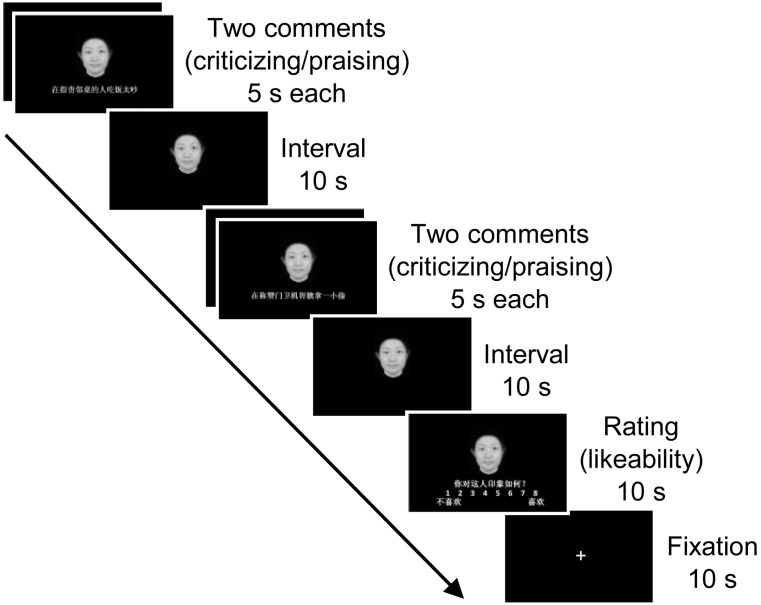
An example of a trial in the affective evaluation task. The individual whose face image is presented here provided written informed consent for the publication of the image.

Faces and comments were presented in a pseudorandom order using E-prime 2.0 software. Four comments were sequentially assigned to one face in order to form an evaluative impression. One third of the faces were paired with criticizing comments, one third praising comments, and the rest were coupled with both criticism and praise (first two criticism, next two praise; or, vice versa). All comments paired with one face remained constant in terms of target (either other people or non-social objects). In each trial of the evaluation task (**Figure 1**), a face-comment combination was presented for 5 s. Successive two face-comment combinations were followed by a 10 s face-alone interval and then another two face-comment combinations. After they learned face-comment pairs participants were shown a scale for 10 s indicating they were required to rate the likeability of the person involved on an 8-point scale (1 = I don’t like the person at all; 8 = I like the person very much). Following a 10 s fixation cross the next trial was initiated.

### Acquisition and Analysis of fMRI Data

During the evaluation task fMRI employing a blood oxygenation level-dependent (BOLD) contrast was conducted on a whole-body 3.0 T MRI scanner (Siemens Trio, Erlangen, Germany) with a 12-channel head coil as signal receiver. Echo planar images were acquired with a gradient-echo planar imaging sequence (TR, 2000 ms; TE, 30 ms; slices, 32; thickness, 4 mm; gap, 0 mm; field of view, 240 mm × 240mm; flip angle, 90°; matrix size, 64 × 64; voxel size, 3.8 mm × 3.8 mm × 4 mm). High-resolution whole-brain structural T1-weighted images were also obtained using a magnetization prepared gradient echo sequence (TR, 1,900 ms; TE, 2.26 ms; thickness,1 mm; sagittal field of view, 256 mm × 256 mm; flip angle, 9°; matrix, 256 × 256 × 176; voxel size, 1 mm × 1 mm × 1 mm) in order to control for any anatomic abnormalities and increase normalization accuracy during fMRI data pre-processing.

fMRI data was preprocessed using DPARSF v2.3 (Data Processing Assistant for Resting-State fMRI software^1^) and analyzed using SPM8 software (Wellcome Trust Centre for Neuroimaging, London, United Kingdom^2^) implemented in Matlab 7 (MathWorks). The first five volumes of each functional time series were discarded to allow for T1 equilibration. Images were corrected for head movement between scans by an affine registration. A two-pass procedure was used, by which images were initially realigned to the first image of the time series and subsequently realigned to the mean of all images. For spatial normalization the mean T1 image of each subject was normalized to the current Montreal Neurological Institute (MNI) template using Dartel. All functional images were hereby transformed into standard MNI space and resampled at 3 mm × 3 mm × 3 mm voxel size. The normalized images were spatially smoothed using an 8-mm FWHM Gaussian kernel.

On the first level, six conditions “criticizing-others,” “praising-others,” “criticizing-objects,” “praising-objects,” “face-alone,” and “rating” were modeled by a stick function convolved with the hemodynamic response function (HRF) (Maldjian et al., 2003). Head movement parameters were included in the design matrix to control for movement related artifacts. On the second level, a paired *t*-test was used to examine the effects of verbal comments vs. face-alone baseline. Due to the absence of gender effects in behavioral results, two-sample *t*-tests were primarily conducted in the second level analysis to confirm, at the neural level, the absence of a main effect of gender on all other experimental manipulations (all face-comment combinations vs. face-alone presentations), and its two-way interactions, respectively, with valence (criticizing vs. praising conditions) and with target (others vs. objects conditions), and three-way interaction with the other two factors [(criticizing- vs. praising-others) vs. (criticizing- vs. praising-objects)]. Subsequent analyses focused on main and interactive effects of valence and target using a flexible factorial ANOVA based on four contrasts (criticizing-others/praising-others/criticizing-objects/praising-objects vs. face-alone). To disentangle significant effects individual parameter estimates were extracted from 8 mm radius spheres centered at the peak coordinates of the effects using MarsBar (Brett et al., 2002).

To further examine the interplay between brain regions underpinning the processing of different comments, a functional connectivity (gPPI) analysis (McLaren et al., 2012) was performed using 8 mm sphere seed regions centered at the peak coordinates of the significant activations in the mPFC, amygdala, pSTS and PCC/precuneus in the prior BOLD response analysis. Here, the coordinates representing the mPFC were the peak of the interactive effect since parameter estimates based on the peaks of both valence and interactive effects showed the same pattern regardless of slightly different coordinates. The data were subjected to flexible factorial ANOVAs followed by parameter estimate extraction using 8 mm radius spheres centered at the peak coordinates of the connectivity effects.

A peak-level family-wise error (FWE) corrected significance threshold of *P* < 0.05 was used on the whole-brain level for all the BOLD response and functional connectivity effects, except the valence × target interaction and amygdala activation in the BOLD response (not connectivity) analysis. Based on our observation of a valence-target interaction in likeability ratings and priori hypothesis for involvement of the amygdala in emotion-laden mentalizing (Mitchell et al., 2005), a more liberal threshold (FWE-corrected *P* < 0.05 adapted to a small-volume correction) was used to identify the potential interaction and modulation of amygdala activation. The small-volume correction was conducted based on the structural mask obtained from the Wake Forest University Pickatlas 3.0 (Tzourio-Mazoyer et al., 2002; Maldjian et al., 2003, 2004). All coordinates are reported in MNI space.

### Statistics

Using IBM SPSS Statistics version 22 behavioral data and parameter estimates extracted from imaging data were analyzed by means of three-way repeated-measures ANOVAs with “comment valence” and “comment target” as within-participants variables and “participant gender” as a between-participants variable. Partial eta squared ($\eta_{p}^{2}$) was calculated as a measure of effect size. The assumption of sphericity was assessed with Mauchly’s test, the Greenhouse-Geisser correction for non-sphericity was applied as required and Bonferroni correction was used when pairwise comparisons were applicable. Pearson correlations between the praise-criticism differences in likeability ratings and extraction of functional connectivity were computed and two-tailed *P*-values were reported. *P* < 0.05 was considered significant in all the analyses.

## Results

### Likeability Ratings

**FIGURE 2:**
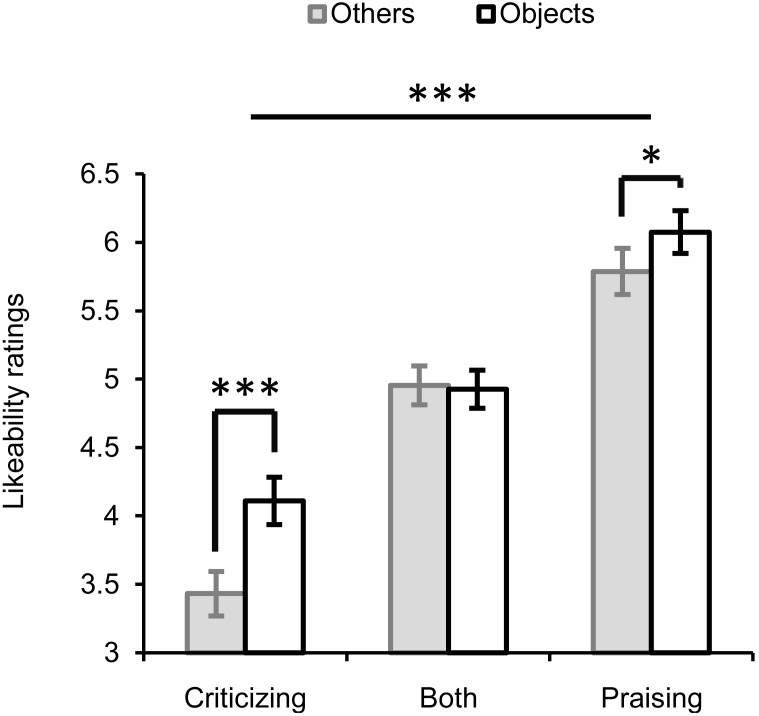
Likeability ratings for faces of individuals making critical, praising, or mixed comments on either others or objects. ^∗^*P* < 0.05, ^∗∗∗^ *P* < 0.001. Bars depict *M* ±*SE*.

The repeated-measures ANOVA with three factors “valence” (praise, criticism or both), “target” (others vs. objects) and “gender” (**Figure 2**) showed significant main effects of valence (*F*_2,56_ = 79.585, *P* < 0.001, $\eta_{p}^{2}$ = 0.74) and target (*F*_1,28_ = 22.416, *P* < 0.001, $\eta_{p}^{2}$ = 0.445). Faces paired with criticism were rated the least likeable (*M* ±*SE*, 3.817 ± 0.149), those paired with praise the most (5.939 ± 0.152), and those paired with both criticism and praise ranked in the middle (4.958 ± 0.124). The overall likeability (for both praise and criticism) of individuals commenting on non-social objects (5.074 ± 0.114) was higher than that of those targeting other people (4.735 ± 0.105). There was a significant interaction between valence and target (*F*_2,56_ = 6.002, *P* = 0.004, $\eta_{p}^{2}$ = 0.177), which was driven by the presence of an others-objects difference in the ratings for faces associated with criticism or praise alone but not for those associated with both criticism and praise. That is, individuals always criticizing or praising others relative to objects were liked less (criticizing: *M*_others_ - *M*_objects_ = -0.711, *F*_1,28_ = 20.317, *P* < 0.001, $\eta_{p}^{2}$ = 0.42; praising: *M*_others_ - *M*_objects_ = -0.289, *F*_1,28_ = 6.482, *P* = 0.017, $\eta_{p}^{2}$ = 0.188). In contrast, the likeability of those who made mixed comments didn’t differ between the social and non-social contexts (*M*_others_ -*M*_objects_ = -0.017, *F*_1,28_ = 0.015, *P* = 0.903, $\eta_{p}^{2}$ = 0.001). No significant main or interactive effects of gender were observed.

### BOLD Responses

**FIGURE 3:**
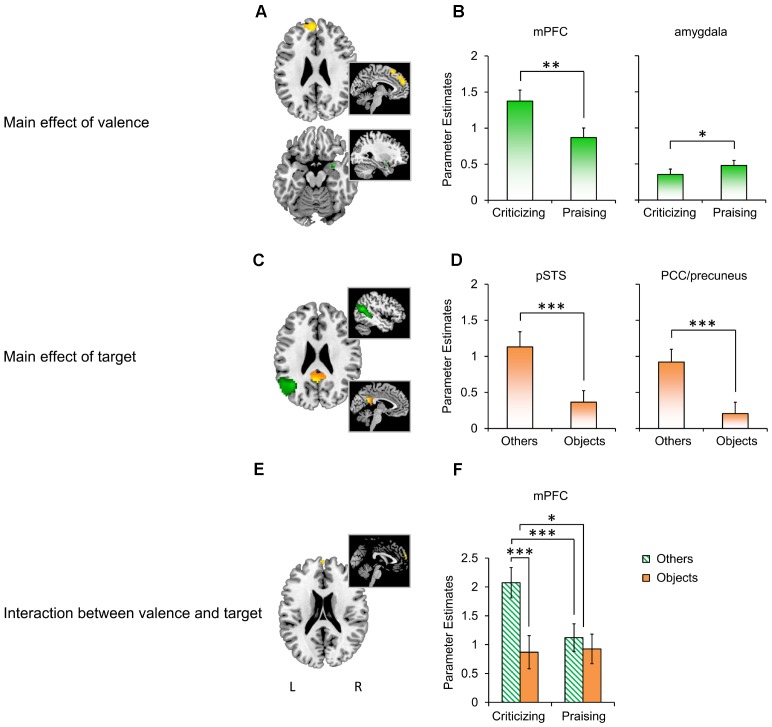
BOLD responses to people- and object-targeted criticism and praise. **(A)** The main effect of valence in the mPFC (yellow; *k* = 205, thresholded at *P* < 0.001 uncorrected for viewing) and right amygdala (green; *k* = 7, *P* < 0.05 uncorrected for viewing). **(B)** Parameter estimates extracted based on the peak of the effect in the mPFC (*x* = –6, *y* = 54, *z* = 27) and amygdala (*x* = 30, *y* = 3, *z* = –18). **(C)** The main effect of target in the left pSTS (green; *k* = 187, *P*_FWE_ < 0.05) and PCC/precuneus (yellow; *k* = 21, *P*_FWE_ < 0.05). **(D)** Extraction based on the peak of the effect in the pSTS (*x* = –45, *y* = –60, *z* = 18) and PCC/precuneus (*x* = –3, *y* = –54, *z* = 21). **(E)** The interaction between valence and target in the mPFC (*k* = 14, *P* < 0.001 uncorrected for viewing). **(F)** Extraction based on the peak of the effect in the mPFC (*x* = 0, *y* = 63, *z* = 24). ^∗^*P* < 0.05, ^∗∗^*P* < 0.01, ^∗∗∗^ *P* < 0.001. Bars depict *M* ± SE. L, left; R, right; mPFC, medial prefrontal cortex; pSTS, posterior superior temporal sulcus; PCC, posterior cingulate cortex.

The whole-brain analysis yielded robust comment-induced activation (**Supplementary Figure S1** and **Supplementary Table S2**) but no main or interactive effects of participant gender. We thus focused on the effects of “valence” and “target” in subsequent analyses. The flexible factorial ANOVA showed a main effect of valence (**Figure 3A**; also see **Supplementary Table S3** for more details) in the left mPFC (*F*_1,87_ = 25.33, *P*_FWE_ = 0.045) and right amygdala (*F*_1,87_ = 10.04, *P*_FWE_ = 0.037), a main effect of target (**Figure 3C**) in the left pSTS (*F*_1,87_ = 37.69, *P*_FWE_ = 0.001) and PCC/precuneus (*F*_1,87_ = 30.9, *P*_FWE_ = 0.007), and their interaction in the mPFC (*F*_1,87_ = 14.67, *P*_FWE_ = 0.050; **Figure 3E**). The extraction of parameter estimates (**Figures 3B,D,F**) revealed stronger reactivity in the mPFC to criticism relative to praise (*M*_criticism_ – *M*_praise_ = 0.506, *F*_1,28_ = 12.698, *P* = 0.001, $\eta_{p}^{2}$ = 0.312), but the opposite pattern for the valence main effect in the right amygdala (*M*_criticism_ -*M*_praise_ = -0.125, *F*_1,28_ = 5.444, *P* = 0.027, $\eta_{p}^{2}$ = 0.163). The main effect of target was confirmed by parameter estimates from the left pSTS (*M*_others_ -*M*_objects_ = 0.765, *F*_1,28_ = 34.505, *P* < 0.001, $\eta_{p}^{2}$ = 0.543) and PCC/precuneus (*M*_others_ -*M*_objects_ = 0.714, *F*_1,28_ = 19.095, *P* < 0.001, $\eta_{p}^{2}$ = 0.405) with both regions displaying enhanced activation in response to comments targeting others as compared to objects. A similar target main effect was also found in the mPFC with greater reactivity to others-targeted relative to object-targeted comments (*M*_others_ -*M*_objects_ = 0.7, *F*_1,28_ = 20.599, *P* < 0.001, $\eta_{p}^{2}$ = 0.424), which did not survive the correction threshold in the previous whole-brain analysis. The extraction further disentangled the interaction between valence and target in the mPFC (*F*_1,28_ = 12.334, *P* = 0.002, $\eta_{p}^{2}$ = 0.306), with pairwise comparisons showing that social criticism produced stronger activation than social praise (*M*_criticism_ – *M*_praise_ = 0.952, *F*_1,28_ = 20.989, *P* < 0.001, $\eta_{p}^{2}$ = 0.428). However, this effect was not observed in the non-social context (*M*_criticism_ -*M*_praise_ = -0.057, *F*_1,28_ = 0.045, *P* = 0.833, $\eta_{p}^{2}$ = 0.002). On the other hand, criticism targeting others, relative to those targeting objects, increased mPFC activation (*M*_others_ -*M*_objects_ = 1.204, *F*_1,28_ = 28.653, *P* < 0.001, $\eta_{p}^{2}$ = 0.506) while the responses to praise did not differ as a function of target (*M*_others_ -*M*_objects_ = 0.195, *F*_1,28_ = 0.998, *P* = 0.326, $\eta_{p}^{2}$ = 0.034).

### Functional Connectivity

**FIGURE 4:**
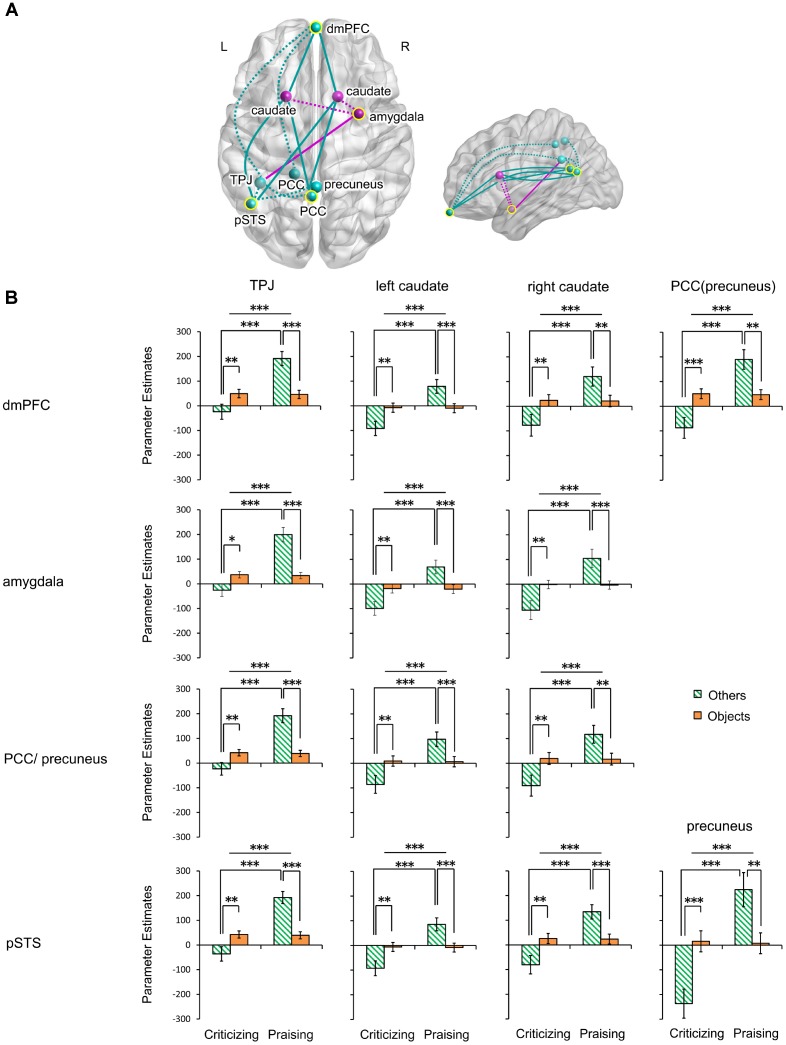
Functional connectivity of the mPFC, right amygdala, PCC/precuneus and left pSTS modulated by people- and object-targeted criticism and praise. **(A)** Axial and sagittal views of altered connections. Yellow circles indicate seed regions. Turquoise, regions and altered connections of seed regions in the mentalizing network; fuchsia, regions and altered connections of seed regions in the affective learning network. Dotted lines, within-network interactions; solid lines, between-network interactions. **(B)** Parameter estimates extracted from the connections. ^∗^*P* < 0.05, ^∗∗^*P* < 0.01, ^∗∗∗^*P* < 0.001. Bars depict *M* ± SE. L, left; R, right; dmPFC, dorsal medial prefrontal cortex; PCC, posterior cingulate cortex; pSTS, posterior superior temporal sulcus; TPJ, temporoparietal junction.

The gPPI analysis (**Table 1** and **Figure 4A**) identified a significant main effect of valence and its interaction with target in the functional connectivity of the mPFC with the left TPJ, bilateral caudate and left PCC including the precuneus. Very similar effects were found in the connectivity of the right amygdala and the PCC/precuneus with the left TPJ and bilateral caudate. These valence main and valence-target interaction effects were also observed in the pSTS coupling with the left TPJ, bilateral caudate and precuneus. No functional connections of the four seed regions showed a significant main effect of target. Parameter estimates extracted from all the significant effects further disentangled the changes in the connections (**Figure 4B** and **Supplementary Table S4**). All the connections were greatly strengthened in response to praise as compared to criticism regardless of target (*P*s < 0.001). All the couplings involving the TPJ were strengthened for others-targeted relative to object-targeted comments (*P*s < 0.05), while no main effect of target was found on the extraction from other connections. Praising comments strengthened the connectivity as compared to critical ones when targeting other people (*P*s < 0.001), although this was not observed in the non-social context. On the other hand, praising people relative to objects strengthened all the connections (*P*s < 0.01). In contrast, when criticizing, targeting others weakened the connections as compared to targeting objects (*P*s < 0.05).

**Table 1:** Significant effects on the connectivity of the mPFC, amygdala, PCC/precuneus and pSTS.

Seeds	Connected regions	Coordinates			Valence		Valence × Target	
		*x*	*y*	*z*	*F*	*P*_FWE_	*F*	*P*_FWE_
mPFC	Left TPJ	-42	-45	27	33.12	0.004	34.8	0.002
	Left caudate	-21	15	15	30.12	0.012	31.54	0.007
	Right caudate	15	15	12	26.74	0.036	27.99	0.024
	Left PCC (precuneus)	-15	-39	45	32.65	0.005	34.18	0.003
amygdala	Left TPJ	-39	-45	30	37.25	0.001	38.96	0.001
	Left caudate	-21	15	15	30.9	0.009	32.07	0.006
	Right caudate	15	15	12	32.19	0.006	33.45	0.004
PCC/	Left TPJ	-39	-45	30	38	0.001	39.82	<0.001
Precuneus	Left caudate	-21	15	15	30.87	0.009	32.21	0.006
	Right caudate	15	15	12	28.98	0.017	30.37	0.011
pSTS	Left TPJ	-42	-48	30	35.66	0.002	36.97	0.001
	Left caudate	-21	15	15	31.01	0.009	32.21	0.006
	Right caudate	15	15	12	32.2	0.006	33.5	0.004
	Precuneus	0	-48	48	26.85	0.035	28.48	0.02

### Correlations Between Neural and Behavioral Results

**FIGURE 5:**
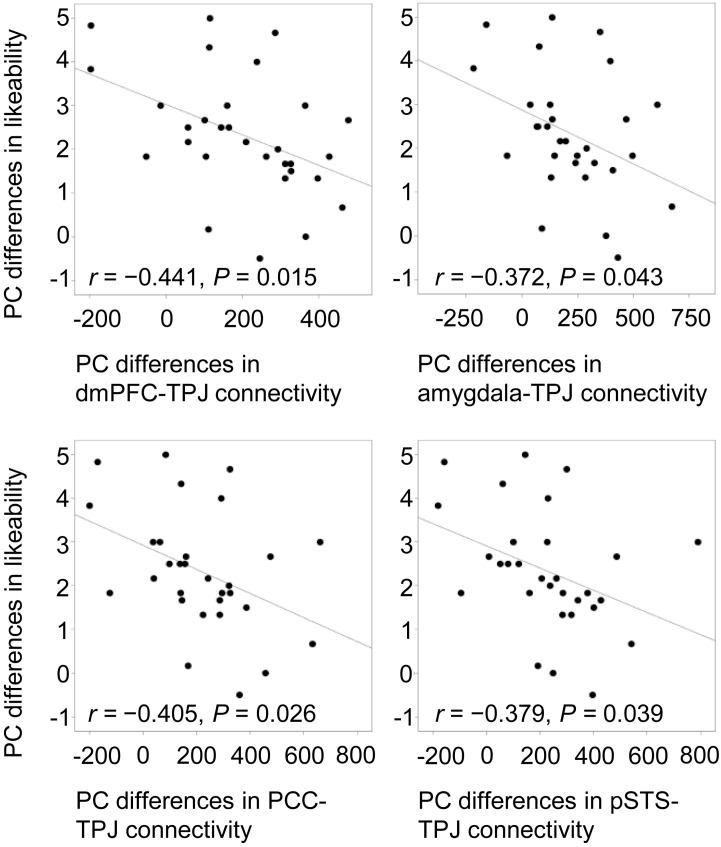
Negative correlations between cross-valence differences in likeability and functional connectivity of the mPFC, right amygdala, PCC and left pSTS with the left TPJ. All cross-valence differences were calculated by subtracting criticism from praise. PC, praise vs. criticism.

To confirm the modulation of likeability by positive and negative verbal cues, Pearson correlations were computed between praise-criticism differences in functional connectivity and differences in ratings. Only in the social condition did the cross-valence differences in ratings correlate negatively with the differences in the connectivity of the four seed regions with the left TPJ (mPFC, *r* = -0.441, *P* = 0.015; amygdala, *r* = -0.372, *P* = 0.043; PCC, *r* = -0.405, *P* = 0.026; pSTS: *r* = -0.379, *P* = 0.039; **Figure 5**), indicating that the larger the social praise-criticism differences in the mPFC-, amygdala-, PCC- and pSTS-TPJ connectivity, the smaller the differences in the likeability ratings.

## Discussion

The present study provides the first evidence for how the brain responds to associating specific individuals with socially and non-socially targeted praise and criticism leading to differential effects on their likeability. Overall, our manipulated verbal cues exert, as hypothesized, markedly distinct effects on neural responses and functional connections involving a network of brain regions supporting mentalizing (mPFC, PCC/precuneus, left pSTS and TPJ) (Frith and Frith, 2006; Mitchell, 2009; Muscatell et al., 2012) and those implicated in affective valuation and approach/avoidance behavior (right amygdala and bilateral caudate).

The activation of the mPFC, largely the dorsal portion, was greatly enhanced in response to individuals associated with criticism, particularly others-targeted criticism, as compared to complimentary counterparts. These valence main and interactive effects suggest a central role of the mPFC in encoding valenced social information during impression formation. Not only has this region been extensively engaged in the processing of social information (Mitchell et al., 2002; Amodio and Frith, 2006), but also this processing is valence-dependent when understanding affective states or making affective judgments of others socially (Harris et al., 2007; Altmann et al., 2012; Perry et al., 2012). On the other hand, using videos showing self-targeted comments, Miedl et al. (2016) failed to identify any praise-criticism differences in the mPFC responses despite strong activation of this region to the valenced cues as compared to neutral ones. Blair et al. (2008) also did not find any valence-specific mPFC responses in healthy individuals to either self- or other-referential praise and criticism, although its reactivity to self-referential criticism differed in individuals with and without generalized social phobia. However, both of these situations primarily involve self-attribution of examples of praising or criticizing characteristics to self or others but not social affective judgments of others as in our study. Thus, the mPFC may differentially encode praising and criticizing comments in the context of evaluating the likeability of others displaying these characteristics.

The activation of other regions of mentalizing, the left pSTS and PCC/precuneus displayed more target-oriented effects. These two regions seem more responsive to different levels of sociality rather than valence-sociality interactions (Schiller et al., 2009; Lahnakoski et al., 2012). While the activation of the mPFC, left pSTS and PCC/precuneus was influenced by different aspects of verbal information manipulation, their functional connectivity changes showed a very similar pattern of modulation by praise and criticism targeting others and objects in terms of the valence main effect and its interaction with target. Indeed, these effects were driven by a decrease of connectivity for social criticism and an increase for social praise. The latter even contributed to the target main effect observed in the connections engaging the left TPJ. The mPFC, TPJ, PCC/precuneus and pSTS have been reported extensively in mentalizing (Frith and Frith, 2006; Mitchell, 2009; Muscatell et al., 2012). Particularly the mPFC, together with the TPJ, are the core regions of the mentalizing network although they may have differentiated functions in trait inferences (Van Overwalle, 2009; Ma et al., 2011). Our findings in connectivity between these regions shed light on the interactions within the mentalizing network in affective evaluation of social others. Here the mPFC, pSTS and PCC/precuneus process valence-dependent social cues via their dynamic interplay with the TPJ. The results may suggest facilitation of making inferences about the individuals involved when they are praising but rejection in understanding them when they are criticizing, particularly in a more socially oriented context.

The right amygdala, unlike the mPFC, was activated less strongly in response to critical relative to praising comments. Both animal and human studies point to reciprocal relations between these two regions, with evidence for them responding inversely, particularly in fearful conditions (Garcia et al., 1999; Quirk et al., 2003; Shin et al., 2005). The valence-induced changes in the mPFC and amygdala here may suggest different but complementary roles of the two regions in learning about the social characteristics of another person. While the amygdala is generally more associated with the processing of negative valence stimuli it can also respond to positive and neutral valence ones. It has consequently been argued that the amygdala is involved more in processing motivational needs and it is this that determines which different valenced stimuli are responded to (Cunningham et al., 2008; Cunningham and Brosch, 2012). Moreover, like the amygdala, the striatum including caudate also plays a critical role in learning emotional and motivational values of both aversive and rewarding stimuli (Delgado et al., 2004; Fareri and Tottenham, 2016). Amygdalostriatal coupling has been implicated in promotion of reward-based valuation and approach behavior (Popescu et al., 2009; Villablanca, 2010; Fareri and Tottenham, 2016). In the current study, functional connectivity between the right amygdala and the bilateral caudate was differentially influenced by valence, being strengthened during exposure to individuals who praise and weakened by ones who criticize. It is possible that strengthened functional connectivity primarily reflects learning of the positive association between praising individuals and social reward, resulting in increased approach behavior. Conversely, weakened connections may indicate reward devaluation and thus avoidance. Taken together, the differential effects of praise and criticism on functional connectivity between the amygdala and TPJ, and additionally between all of the mentalizing regions and the bilateral caudate, further suggest that the mentalizing and affective processing networks are interacting to facilitate learning of both positive and negative associations based on trait inferences, guiding subsequent social preferences and behavioral adaptation.

Interestingly, the differences in functional connectivity strengths between praise and criticism conditions within the mentalizing regions and between the TPJ and amygdala were negatively correlated with corresponding differences in likeability ratings. That is, the larger the cross-valence differences in the functional connections, the smaller such differences were in likeability ratings. This may suggest that the interplay within regions in the mentalizing network and between them and the amygdala may contribute to how much valenced social cues inform affective judgments. In other words, this provides further evidence that interactions between the metalizing and reward learning networks underlie affective evaluation of social others cued by praise and criticism. And in this interactive process, the TPJ may function as a hub connecting these networks, which consists with evidence from brain-damaged patients indicating a role of the TPJ as a necessary mediator of social inference (Samson et al., 2004) and extends this role into emotional and motivational social inference.

In line with the fMRI findings likeability ratings for individuals associated with praise and criticism showed significant main and interactive effects of both valence and target, suggesting the role of verbal cues in biasing affective judgments. The neutral faces of individuals associated with negative attributes (critical toward others/objects) were robustly evaluated as less likeable compared to those associated with positive traits (praising/prosocial). In particular, individuals who criticized others were liked even less than those critical of objects. It is notable that even though the critical comments used in the present study are not extreme, and highly likely to be made in everyday, real-life social settings, they nevertheless still have a strong negative impact on how individuals producing them are judged.

Although some of our fMRI results displayed enhanced brain responses to praise of others, this unexpectedly did not result in individuals associated with them being liked more than ones associated with object-targeted praise. It is possible that individuals who constantly praise other people may be viewed as trying deliberately to please them, and this could act to devalue its positive impact. Indeed, this is supported by previous findings in a binocular rivalry task that visual dominance did not differ between faces previously paired with positive behaviors involving others relative to objects although this person-object difference was observed in faces associated with negative behaviors (Anderson et al., 2011). Praising evaluations are also not always positive for everyone and can evoke fearful responses. Indeed, a recent study hypothesized that while social criticism hurts everybody, responses to social praise may be heterogeneous due to variant levels of fear for praise (Miedl et al., 2016). Our findings in this regard may indicate that person-directed praise is more complicated than criticism in terms of understanding its intention or making inferences based upon it.

We did not observe gender-specific effects of our manipulated verbal cues at either neural or behavioral levels. Possibly gender differences would occur in heterosexual interactions. Given the complexity of our experiment design, however, further analysis of potential heterosexual interaction effects were not really allowed in respect of statistical power. The sex of both comment makers (faces) and receivers (participants) should both be considered in future work.

## Conclusion

Using naturalistic verbal comments the present study demonstrates that in both men and women encoding faces paired praising and criticizing cues involves person inferences and associative learning supported by interactions within and between mentalizing and affective processing networks, which facilitate social preferences and social approach or avoidance decisions in future interpersonal interactions. In social communication therefore, caution should be exercised when evaluative comments, particularly negative ones, are made about others.

## Author Contributions

SG, YG, JL, and YZ collected the data. SG, YG, and SY analyzed the data. SG and SY conceived the study, interpreted the results, and wrote the paper. All authors discussed the results and commented on the manuscript.

## Conflict of Interest Statement

The authors declare that the research was conducted in the absence of any commercial or financial relationships that could be construed as a potential conflict of interest.

## Footnotes

1 http://www.restfmri.net/forum/DPARSF

2 http://www.fil.ion.ucl.ac.uk/spm
