# Supplementary material for: Encoding Praise and Criticism During Social Evaluation Alters Interactive Responses in the Mentalizing and Affective Learning Networks
Source: Front Neurosci. 2018 Sep 4;12:611. doi: 10.3389/fnins.2018.00611 (PMC6131607; doi:10.3389/fnins.2018.00611)
Supplement: Supplementary file 1 [file Table_1.docx]

Supplementary Material

Encoding praise and criticism during social evaluation alters interactive responses in the mentalizing and affective learning networks

Shan Gao, Yayuan Geng, Jia Li, Yunxiao Zhou, Shuxia Yao^*^

*** Correspondence:** [yaoshuxia12@126.com](mailto:yaoshuxia12@126.com)

**Table S1. Ratings for the four categories of comments (*M* ± SD)**

|  | Categories of Comments | | | |
| --- | --- | --- | --- | --- |
| Variables | Criticizing-Others | Praising-Others | Criticizing-Objects | Praising-Objects |
| Valence | 3.608 ± 1.086 | 6.763 ± 0.837 | 3.653 ± 1.090 | 6.667 ± 0.897 |
| Arousal | 6.432 ± 1.249 | 6.397 ± 1.357 | 6.420 ± 1.165 | 6.293 ± 1.361 |
| Likelihood | 7.134 ± 0.745 | 7.247 ± 0.788 | 7.204 ± 0.754 | 7.260 ± 0.771 |
| Comprehension | 7.526 ± 0.914 | 7.650 ± 0.770 | 7.579 ± 0.818 | 7.605 ± 0.852 |
