# Supplementary material for: Encoding Praise and Criticism During Social Evaluation Alters Interactive Responses in the Mentalizing and Affective Learning Networks
Source: Front Neurosci. 2018 Sep 4;12:611. doi: 10.3389/fnins.2018.00611 (PMC6131607; doi:10.3389/fnins.2018.00611)
Supplement: Supplementary file 2 [file Table_2.docx]

Supplementary Material

Encoding praise and criticism during social evaluation alters interactive responses in the mentalizing and affective learning networks

Shan Gao, Yayuan Geng, Jia Li, Yunxiao Zhou, Shuxia Yao^*^

*** Correspondence:** [yaoshuxia12@126.com](mailto:yaoshuxia12@126.com)

**Table S2. Comments > face-alone baseline at the whole-brain level**

| Brain regions | Side | MNI coordinates | | | Cluster size | Peak-level |
| --- | --- | --- | --- | --- | --- | --- |
|  |  | *x* | *y* | *z* | *k* | *t* |
| Lingual Gyrus | L | −21 | −96 | −9 | 5044 | 17.93 |
| Lingual Gyrus | R | 12 | −81 | −3 |  | 17.04 |
| Lingual Gyrus | R | 18 | −90 | −12 |  | 16.58 |
| Superior frontal gyrus | L | −6 | 15 | 63 | 580 | 14.34 |
| Medial temporal gyrus | L | −9 | 39 | 45 |  | 11.94 |
| Superior frontal gyrus | L | −9 | 57 | 27 |  | 11.60 |
| Inferior frontal gyrus | L | −42 | 24 | −12 | 1102 | 12.97 |
| Middle frontal gyrus | L | −42 | 0 | 51 |  | 12.36 |
| Inferior frontal gyrus | L | −48 | 30 | −3 |  | 12.22 |
| Cerebellum | M | 3 | −54 | −39 | 107 | 11.90 |
| Parahippocampal gyrus | R | 33 | −9 | −15 | 86 | 11.55 |
| Amygdala | R | 33 | −3 | −24 |  | 9.56 |
| Parahippocampal gyrus | R | 21 | −6 | −18 |  | 8.15 |
| Temporal pole | R | 45 | 21 | −21 | 122 | 10.80 |
| Middle frontal gyrus | R | 42 | 33 | −15 |  | 10.16 |
| Inferior frontal gyrus | R | 33 | 27 | −15 |  | 9.77 |
| Middle frontal gyrus | R | 51 | 30 | 33 | 99 | 10.65 |
| Inferior frontal gyrus | R | 57 | 30 | 15 |  | 8.19 |
| Caudate | R | 18 | −9 | 21 | 33 | 10.09 |

Height threshold: *t* = 7.4, *P*_FWE_ < 0.001; extent threshold: *k* = 30 voxels. L, left; M, middle; R, right.
