# Supplementary material for: Encoding Praise and Criticism During Social Evaluation Alters Interactive Responses in the Mentalizing and Affective Learning Networks
Source: Front Neurosci. 2018 Sep 4;12:611. doi: 10.3389/fnins.2018.00611 (PMC6131607; doi:10.3389/fnins.2018.00611)
Supplement: Supplementary file 3 [file Table_3.docx]

Supplementary Material

Encoding praise and criticism during social evaluation alters interactive responses in the mentalizing and affective learning networks

Shan Gao, Yayuan Geng, Jia Li, Yunxiao Zhou, Shuxia Yao^*^

*** Correspondence:** [yaoshuxia12@126.com](mailto:yaoshuxia12@126.com)

**Table S3. Significant effects in the whole-brain ANOVA with the factors valence (criticizing vs. praising) and target (others vs. objects)**

| Brain regions | MNI coordinates | | | Cluster size | Peak-level | |
| --- | --- | --- | --- | --- | --- | --- |
|  | *x* | *y* | *z* | *k* | *F* | *P*_FWE_ |
| **Main effect of valence** |  |  |  |  |  |  |
| Superior temporal gyrus | −51 | −12 | −27 | 7 | 28.34 | 0.016 |
| Superior frontal gyrus | −6 | 54 | 27 | 6 | 25.33 | 0.045 |
| amygdala | 30 | 3 | −18 | 5 | 10.04 | 0.039 |
| **Main effect of target** |  |  |  |  |  |  |
| Cerebellum | 24 | −78 | −33 | 47 | 38.61 | 0.001 |
| Superior temporal gyrus | −45 | −60 | 18 | 187 | 37.69 | 0.001 |
| Superior temporal gyrus | −57 | −51 | 9 |  | 31.80 | 0.005 |
| Middle temporal gyrus | −51 | −42 | 3 |  | 30.78 | 0.007 |
| PCC/ Precuneus | −3 | −54 | 21 | 21 | 30.90 | 0.007 |
| **Valence × target interaction** |  |  |  |  |  |  |
| Medial frontal gyrus | 0 | 63 | 24 | 5 | 14.67 | 0.050 |

Height threshold: *F* = 25.03, *P*_FWE_ < 0.05 except for the amygdala activation and valence × target interaction, which were adapted to the threshold of *P*_FWE_ < 0.05 based on small-volume correction.
