# Supplementary material for: Encoding Praise and Criticism During Social Evaluation Alters Interactive Responses in the Mentalizing and Affective Learning Networks
Source: Front Neurosci. 2018 Sep 4;12:611. doi: 10.3389/fnins.2018.00611 (PMC6131607; doi:10.3389/fnins.2018.00611)
Supplement: Supplementary file 4 [file Table_4.docx]

Supplementary Material

Encoding praise and criticism during social evaluation alters interactive responses in the mentalizing and affective learning networks

Shan Gao, Yayuan Geng, Jia Li, Yunxiao Zhou, Shuxia Yao^*^

*** Correspondence:** [yaoshuxia12@126.com](mailto:yaoshuxia12@126.com)

**Table S4. Significant effects on parameter estimates extracted from altered connections**

| Seeds | Connected Regions | Valence | | Target | | Valence × Target | |  |
| --- | --- | --- | --- | --- | --- | --- | --- | --- |
|  |  | *F* | *P* | *F* | *P* | *F* | *P* | |
| mPFC | left TPJ | 33.035 | <0.001 | 4.45 | 0.044 | 32.149 | <0.001 | |
|  | left caudate | 34.818 | <0.001 |  |  | 37.059 | <0.001 | |
|  | right caudate | 27.297 | <0.001 |  |  | 28.998 | <0.001 | |
|  | left PCC (precuneus) | 32.214 | <0.001 |  |  | 33.831 | <0.001 | |
| amygdala | left TPJ | 31.212 | <0.001 | 11.092 | 0.002 | 32.956 | <0.001 | |
|  | left caudate | 37.682 | <0.001 |  |  | 39.443 | <0.001 | |
|  | right caudate | 28.568 | <0.001 |  |  | 29.638 | <0.001 | |
| PCC/ | left TPJ | 32.557 | <0.001 | 7.86 | 0.009 | 34.539 | <0.001 | |
| precuneus | left caudate | 35.159 | <0.001 |  |  | 37.244 | <0.001 | |
|  | right caudate | 25.487 | <0.001 |  |  | 26.735 | <0.001 | |
| pSTS | left TPJ | 35.164 | <0.001 | 5.56 | 0.026 | 37.292 | <0.001 | |
|  | left caudate | 38.736 | <0.001 |  |  | 40.809 | <0.001 | |
|  | right caudate | 29.67 | <0.001 |  |  | 30.001 | <0.001 | |
|  | precuneus | 29.229 | <0.001 |  |  | 30.685 | <0.001 | |
