# Supplementary material for: Encoding Praise and Criticism During Social Evaluation Alters Interactive Responses in the Mentalizing and Affective Learning Networks
Source: Front Neurosci. 2018 Sep 4;12:611. doi: 10.3389/fnins.2018.00611 (PMC6131607; doi:10.3389/fnins.2018.00611)
Supplement: Supplementary file 5 [file Image_1.PDF]

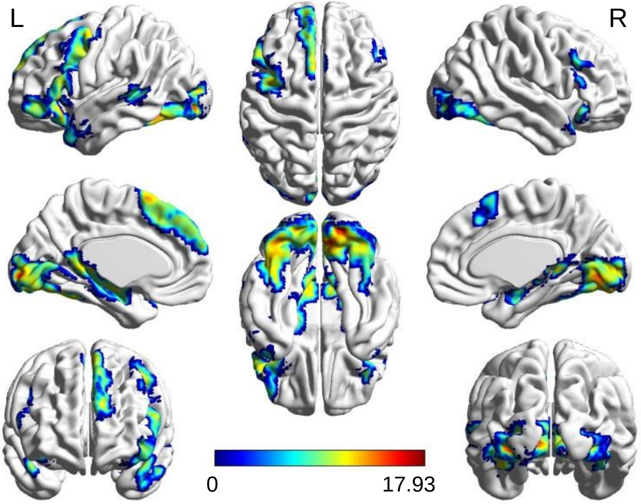

Figure S1. The *t*-map of comments > face-alone contrast. This effect was basically found in the anterior regions including the mPFC, precentral gyrus, orbital frontal cortex and temporal pole, in the sub-cortical regions including the amygdala and caudate and in the visual cortex.
